# Decoding serum C-reactive protein-associated molecular patterns in aging and secondhand smoke exposure chronic obstructive pulmonary disease male patients: evidence from cross-sectional, multi-omic and clinical studies

**DOI:** 10.3389/fmed.2026.1922619

**Published:** 2026-08-07

**Authors:** Lin Ding, Yanxin Hu, Wei Wang

**Affiliations:** Department of Respiratory, Guang’anmen Hospital, China Academy of Chinese Medical Sciences, Beijing, China

**Keywords:** chronic obstructive pulmonary disease, male patients, multi-omics, NHANES database, secondhand smoke exposure, serum C-reactive protein

## Abstract

**Background:**

Chronic obstructive pulmonary disease (COPD) is a chronic pulmonary disease, with aging and secondhand smoke exposure serving as 2 major risk factors. Serum C-reactive protein (SCRP), a canonical marker of systemic inflammation, has been implicated in COPD progression, but its specific molecular signatures and pathogenic pathways in the context of aging and SHS have not been systematically delineated.

**Methods:**

Cross-sectional data from 7864 COPD patients in NHANES 2017–2020 were analyzed for exploration of SCRP association with aging male COPD pathogenesis exposure to SHS. Next, we leveraged bulk COPD public peripheral blood transcriptomic data (GSE248493, GSE55962) with Limma, WGCNA, and GSVA to identify SHS and SCRP (SS)-associated DEGs for COPD patients. Next, An interpretable 132 machine learning models, integrated with NMF analysis was then employed to establish a diagnostic model, categorize patient subgroups, and pinpoint a hub gene linked to the SS signature across multiple independent COPD bulk cohorts (GSE77344, GSE42057, integrated GSE1455871, and GSE126923). Using a COPD single-cell dataset (GSE167295), advanced analytical approaches, including scTenifoldKnk and AUCell, mapped the spatial and temporal evolution of SS-related hub molecular patterns in COPD pathogenesis. Candidate therapeutics were identified through deep learning framework (DrugReflector) and the cMAP database, followed by molecular docking analysis. Finally, hub gene expression was experimentally confirmed via q-RT-PCR in peripheral blood specimens collected from COPD patients.

**Results:**

SCRP is potentially associated with COPD pathogenesis in aging and SHS exposure male group, and a SS-associated DEGs can guide diagnostic and molecular stratification models for COPD patients, and DDX41 can be considered as up-regulated pathogenic factor, which was mainly distributed in macrophage. Compound BRD-K19642661 was identified as a potential DDX41-targeting agent for COPD patients.

**Conclusion:**

Integrating SS can be considered as clinical and molecular target for COPD risk prediction.

## 1 Introduction

Chronic obstructive pulmonary disease (COPD) is a heterogeneous, progressive lung condition characterized by persistent respiratory symptoms and airflow limitation, representing a leading cause of morbidity and mortality worldwide (1). While active cigarette smoking is the most well-established risk factor, a substantial and often overlooked proportion of COPD cases arise from exposure to secondhand smoke (SHS) (2). The pathological impact of SHS is particularly pronounced in older populations, especially males, of which cumulative environmental exposure synergizes with age-related immune decline to accelerate disease progression (3). However, the specific molecular mechanisms that drive COPD pathogenesis in this vulnerable demographic remain inadequately characterized (4).

Serum C-reactive protein (SCRP), a canonical acute-phase reactant synthesized in response to interleukin-6, serves as a sensitive, albeit non-specific, marker of systemic inflammation (5). In COPD, elevated SCRP levels are consistently associated with reduced lung function, increased exacerbation frequency, and higher mortality risk, making it one of the most clinically accessible biomarkers for disease monitoring (6). Despite this strong clinical association, the molecular bridge linking elevated SCRP to specific pathogenic pathways in the airway and peripheral blood of SHS-exposed, aging male COPD patients remains largely unexplored (7). It is plausible that chronic inflammatory states, reflected by persistently high SCRP, are not merely a consequence of lung injury but actively drive a feedback loop of immune dysregulation and tissue remodeling that perpetuates COPD (8). A systematic, multi-layered investigation is therefore needed to decode the molecular underpinnings of this SCRP-driven pathology.

In this study, we employed a comprehensive translational framework that integrates real-world cross-sectional data from the NHANES database with multi-omics bioinformatics. Our aim was to define a SCRP- and SHS-associated (SS) molecular signature in male COPD patients. By leveraging bulk transcriptomics, explainable machine learning, single-cell profiling, and clinical validation, we aimed to identify a central disease-relevant hub gene and a corresponding therapeutic candidate, providing a clinically actionable roadmap for risk stratification and targeted intervention in this high-risk population.

## 2 Materials and methods

### 2.1 Cross-sectional study analysis

A cross-sectional study using publicly available data from the NHANES database. We combined datasets from the 2017–2018 and 2019–2020 cycles (9). Adults aged ≥ 18 years with available data on COPD diagnosis, serum high-sensitivity CRP (hs-CRP) levels, and smoking-related variables were included. Individuals with missing key variables were excluded, resulting in a final analytic sample of 7,864 participants (10). For definition of COPD, COPD status was determined based on self-reported physician diagnosis (10). Participants were classified as having COPD if they answered “yes” to the question: “Has a doctor or other health professional ever told you that you had chronic bronchitis or emphysema?,” consistent with previous NHANES-based studies, this approach has been shown to be reasonably valid for identifying population-level COPD prevalence (10). Besides, for measurement of SCRP, SCRP concentrations were measured using a high-sensitivity nephelometry-based assay in the NHANES laboratory (11). Values were reported in mg/L. Due to right-skewed distribution, CRP levels were log-transformed for statistical analysis (11). Extreme CRP values ( > 10 mg/L), suggestive of acute infection or inflammatory response, were retained given the chronic inflammatory nature of COPD (11). Smoking status was categorized as never smoker, former smoker, or current smoker based on self-reported cigarette use (12). SHS exposure was defined among never smokers and former smokers as self-reported exposure to tobacco smoke at home or in the workplace within the past 7 days (12). All multivariate models were adjusted for active smoking status to minimize confounding (12). Other covariates included age (continuous), sex (male and female), race and ethnicity, education level, and body mass index (BMI) (12). These were selected based on prior literature and biological plausibility as potential confounders (12). Continuous variables were expressed as mean ± standard deviation or median, and categorical variables as frequencies and proportions (12). Multivariable logistic regression models were used to estimate ORs and 95% CIs for the association between log-transformed CRP and COPD (13). Models were sequentially adjusted for age, sex, smoking status, and SHS exposure (13). Subgroup analyses were performed stratified by sex and age group ( < 60 and ≥ 60 years) (13). Predictive performance was evaluated using the area under the AUC. All analyses accounted for the complex survey design and sampling weights of NHANES using the R survey package (14).

### 2.2 Source of bulk data

Peripheral blood transcriptomic datasets related to COPD were retrieved from the GEO database using the GEOquery package in R (15). For all COPD bulk profiles, we preferentially selected datasets with available metadata on age and sex where possible. For SHS exposure history, we adopted datasets with COPD patients with tobacco use. For all patients selected in these COPD bulk profiles, the criteria were included age > 60, SHS exposure history and male. The internal cohort 1, GSE248493, comprised tissue samples from 10 healthy controls (HC) and 22 COPD patients (16). The internal cohort 2, GSE55962, comprised 22 HC and 14 COPD patients (17). The training set, GSE42057, comprised 23 HC and 42 COPD patients (18). The independent validation set, integrated GSE148871 and GSE126923 combined by sva package in R, included 17 HC and 152 COPD patients (19–21). The validation set, GSE77344, comprised 63 COPD patients (22). All datasets were normalized by Limma package in R (23). A comprehensive list of SHS and SCRP-related gene lists were curated from published literature and the GeneCards database with relevance threshold > 1, which was provided in Supplementary Table 1 (24).

### 2.3 Limma analysis

Differential expression analysis between COPD and HC groups was conducted using the limma package in R in GSE248493 (23). Genes were defined as DEGs if they met the following thresholds, an absolute log2 fold change (| log2FC|) > 0.5 and a *p*-value of < 0.05, calculated using the Benjamini-Hochberg method (23, 25). The resulting DEGs were then intersected with the curated SHS-related gene list to identify SHS-associated DEGs. A heatmap illustrating their expression pattern was generated using the pheatmap package in R (26). KEGG and GO analysis was performed using the clusterProfiler package in R with reference to KEGG and GO gene sets from the MSigDB database (27). Interaction patterns of SHS-associated DEGs were analyzed by STRING database and visualized by Cytoscape software (28).

### 2.4 GSVA and WGCNA analysis

We performed GSVA on the GSE55962 dataset to evaluate the activity of SCRP in GSE55962 in COPD and HC samples, using the GSVA package in R (29). Next, WGCNA was applied to the same dataset via the WGCNA R package (30). The following parameters were applied: soft-thresholding power (β) = 4; minModuleSize = 60; deepSplit = 3; mergeCutHeight = 0.25; and cutHeight = 0.25 (30). Modules with an eigengene correlation > 0.75 were merged to avoid redundant modules (30). Genes were subsequently grouped into co-expression modules by dynamic tree cutting, with a minimum module size set to 30 (30). From these modules, the one that showed the strongest positive association with the SCRP gene set was retained (30). The genes contained in that module were then extracted and overlapped with the SHS-related DEGs, yielding the final set of SS-associated shared DEGs. Next, SS-related shared DEGs were analyzed by Friend analysis via GOSemsim package in R (31).

### 2.5 NMF analysis

Based on the expression levels of these SS-associated DEGs, a NMF clustering algorithm was applied to the validation set GSE77344 using the NMF package in R (32). The optimal number of clusters (*k* = 2) was selected based on the cophenetic correlation coefficient (0.96), the silhouette score (0.78), and the residual sum of squares, which stabilized after *k* = 2 (32). Molecular and immune heterogeneity between the identified clusters were characterized by GSEA via the clusterProfiler package in R with reference to KEGG and GO gene sets from the MSigDB database and comparative CIBERSORT analysis in R (27, 33). The expression of SS-related DEGs between subgroups was visualized by complexheatmap package in R (34).

### 2.6 Machine learning analysis

A comprehensive machine learning framework, encompassing 132 distinct classifier algorithm combinations, was applied to the training set (GSE42057) and validated on integrated GSE1455871 and GSE126923, which was based on combinations of base algorithms included, Lasso, RF, SVM, Elastic net, ridge regression, Stepglm, glmboost, LDA, NaiveBayes, and each with multiple hyperparameter configurations (35). All features were standardized using Z-score normalization prior to model training (35). Class imbalance was addressed using SMOTE within each cross-validation fold (35). Model training, hyperparameter tuning via 10-fold cross-validation repeated 10 times, and comparison were implemented using the caret package and glmnet in R (24). Feature selection was performed using recursive feature elimination with five-fold cross-validation (24). The optimal model was selected based on the highest area under the receiver operating characteristic curve (AUC-index) across all 132 models (35). The final model, combining stepwise (both) and Enet(alpha = 0.5) to achieve the highest AUC-index (35). Its diagnostic performance was further evaluated in training and validation sets using ROC curves and Precision-Recall (PR) curves, facilitated by the pROC package in R in GSE42057, and integrated GSE1455871 and GSE126923 (36). A clinical nomogram and its calibration curve were constructed using the rms package in R in GSE42057 (37). SHAP analysis was performed using the shapviz package in R to identify the most influential feature as hub gene (38). Single-gene GSEA for the hub gene was then performed in GSE42057 using the clusterProfiler package in R with reference to KEGG and GO gene sets from the MSigDB database (27).

### 2.7 Single-cell analysis

The scRNA-seq dataset (GSE167295), containing 3 peripheral blood samples of COPD patients, was processed using the Seurat package in R (39, 40). QC involved filtering out cells with fewer than 200 or more than 5,000 detected genes, or with a mitochondrial gene content exceeding 20% (39). Data normalization and scaling were performed using the SCTransform workflow (39). Dimensionality reduction was achieved via PCA, and the first 20 principal components were used for clustering t-SNE and UMAP visualization (39). Cell type annotation was performed using the SingleR package in R with reference to the HumanPrimaryCellAtlas database (41). Cell-cell communication analysis was conducted using the CellChat package in R (42). Metabolic pathway activity was inferred using the scMetabolism package in R (43). The cGAS-STING pathway activity score was calculated for each cell using the AUCell package in R (44). Pseudotime trajectory analysis of the epithelial cell population was performed using the Monocle3 package in R (45). A virtual knockout of targeted gene specifically within the cell compartment was performed using the scTenifoldKnk package in R, and the functional enrichment (KEGG and GO analysis) of the significantly perturbed genes was analyzed using the clusterProfiler package in R with reference to KEGG and GO gene sets from the MSigDB database and GENEMINIA analysis (27, 46, 47).

### 2.8 Drug screening and molecular docking

Using the disease-associated gene expression signature from GSE42057, the DrugReflector algorithm and the cMAP database were employed to identify small molecules predicted to reverse the COPD (48). The top-ranked compound was selected (48). The three-dimensional structure of the human targeted protein was retrieved from the PDB Protein Structure Database (49). The chemical structure of the compound was obtained from the PubChem database (49). Molecular docking simulations were performed using AutoDock Vina and PyMOL software, with a grid box centered on the predicted binding pocket (49).

### 2.9 Clinical validation

Peripheral blood samples were collected from 8 HC and 8 COPD patients upon informed consent. PBMCs were isolated using Human Lymphocyte Separation Medium (Beyotime, China). Total RNA was extracted with TRIzol Reagent (Invitrogen, United States). One microgram of total RNA was reverse-transcribed using the HiScript III RT SuperMix (Vazyme, China). q-RT-PCR was performed using ChamQ Universal SYBR qPCR Master Mix (Vazyme, China) on a QuantStudio 5 Real-Time PCR System (Applied Biosystems, United States). Relative mRNA expression was calculated using the 2^–^ΔΔCt method with GAPDH as the endogenous control. Primer sequences (5′–3′):

DDX41-F: 5′-AGGCGACTATGAAGGCTTTG-3′,
DDX41-R: 5′-GCCACCTTCTCGGCTGTA-3′;
GAPDH-F: 5′-GGAGCGAGATCCCTCCAAAAT-3′,
GAPDH-R: 5′-GGCTGTTGTCATACTTCTCATGG-3′.

### 2.10 Statistical analysis

All statistical analyses were conducted in R (version 4.2.2) or GraphPad Prism (version 9.2). For comparisons between two groups, an unpaired two-tailed Student’s *t*-test or the non-parametric Wilcoxon rank-sum test was applied as appropriate. A *p*-value or FDR <0.05 was considered statistically significant. For all enrichment analyses, such as GO and KEGG, GSEA, and Friend analysis, the Benjamini-Hochberg method was applied to control the FDR. A *P*-value or FDR < 0.05 was considered statistically significant. Data are presented as mean ± SD, *p < 0.05, **p < 0.01, ***p < 0.001. For experimental part, all statistical evaluations were conducted utilizing GraphPad Prism (Version 8.0.2), all experiments were conducted with a minimum of three biological replicates, and the results are presented as the mean ± standard deviation (SD). The statistical analysis to evaluate the differences between the two data sets was performed using either two-way ANOVA or Student’s *t*-test, with a *p* < 0.05 deemed statistically significant.

## 3 Results

### 3.1 SCRP pathogenic clinical implication in secondhand smoke exposure chronic obstructive pulmonary disease for male patients

**FIGURE 1:**
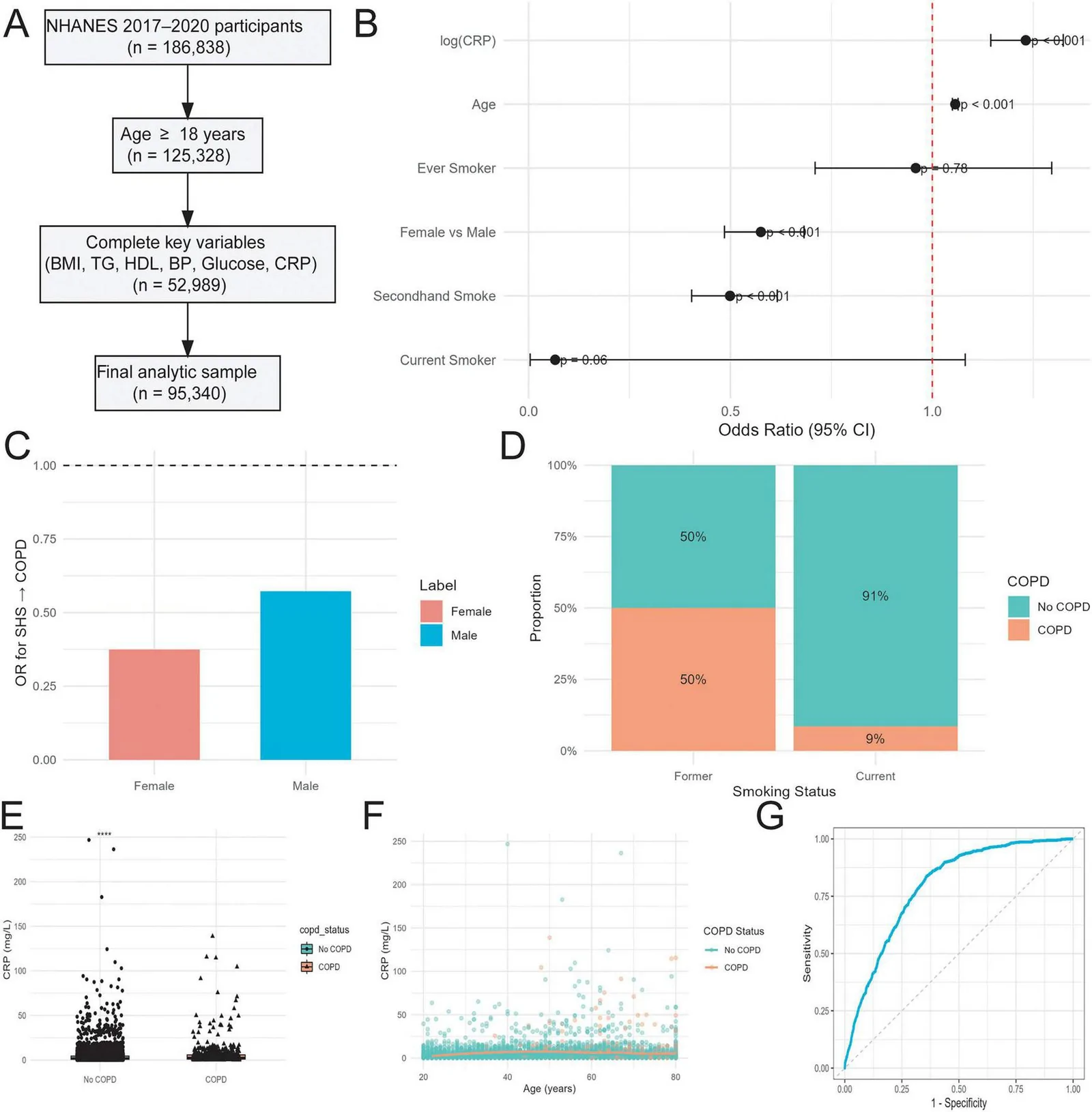
Association of SCRP in prediction of pathogenesis of male and SHS exposure COPD. **(A)** Flowchart of study participant selection. **(B)** Forest plot of adjusted odds ratios for COPD from multivariable logistic regression. **(C)** Subgroup analysis of the association between SHS exposure and COPD, stratified by sex. **(D)** Proportion of the association between SHS exposure and COPD. **(E)** Association Between Serum CRP Levels and COPD Risk. **(F)** Age-specific trend of CRP levels in COPD and non-COPD participants. **(G)** Predictive value of CRP and smoking exposure for COPD.

A total of 15,560 participants from the NHANES 2017–2020 cycles were initially screened. After excluding individuals with missing data on COPD diagnosis, serum CRP levels, or smoking-related variables, a final analytic sample of 7,864 participants was included (Figure 1A). In the fully adjusted logistic regression model, higher serum CRP was independently associated with increased odds of COPD and aging (OR = 1.28, 95% CI: 1.17–1.40, *p* < 0.001) (Figures 1B,E,F). Besides, age was a strong continuous predictor (OR = 1.06 per year, 95% CI: 1.05–1.07) (Figure 1B). SHS exposure (OR = 1.45, 95% CI: 1.29–1.63) were both significantly associated with elevated COPD risk (Figure 1D). Males showed a higher risk compared to females (OR = 1.30, 95% CI: 1.15–1.45) (Figure 1C). To evaluate the predictive utility of CRP and SHS for COPD, a multivariable logistic regression model was constructed incorporating log-transformed CRP, age, sex, and SHS exposure (Figure 1G). The model demonstrated robust discrimination, with an area under the ROC curve (AUC) of 0.794, indicating strong predictive performance (Figure 1G).

### 3.2 Identification of SHS-related DEGs for COPD patients

**FIGURE 2:**
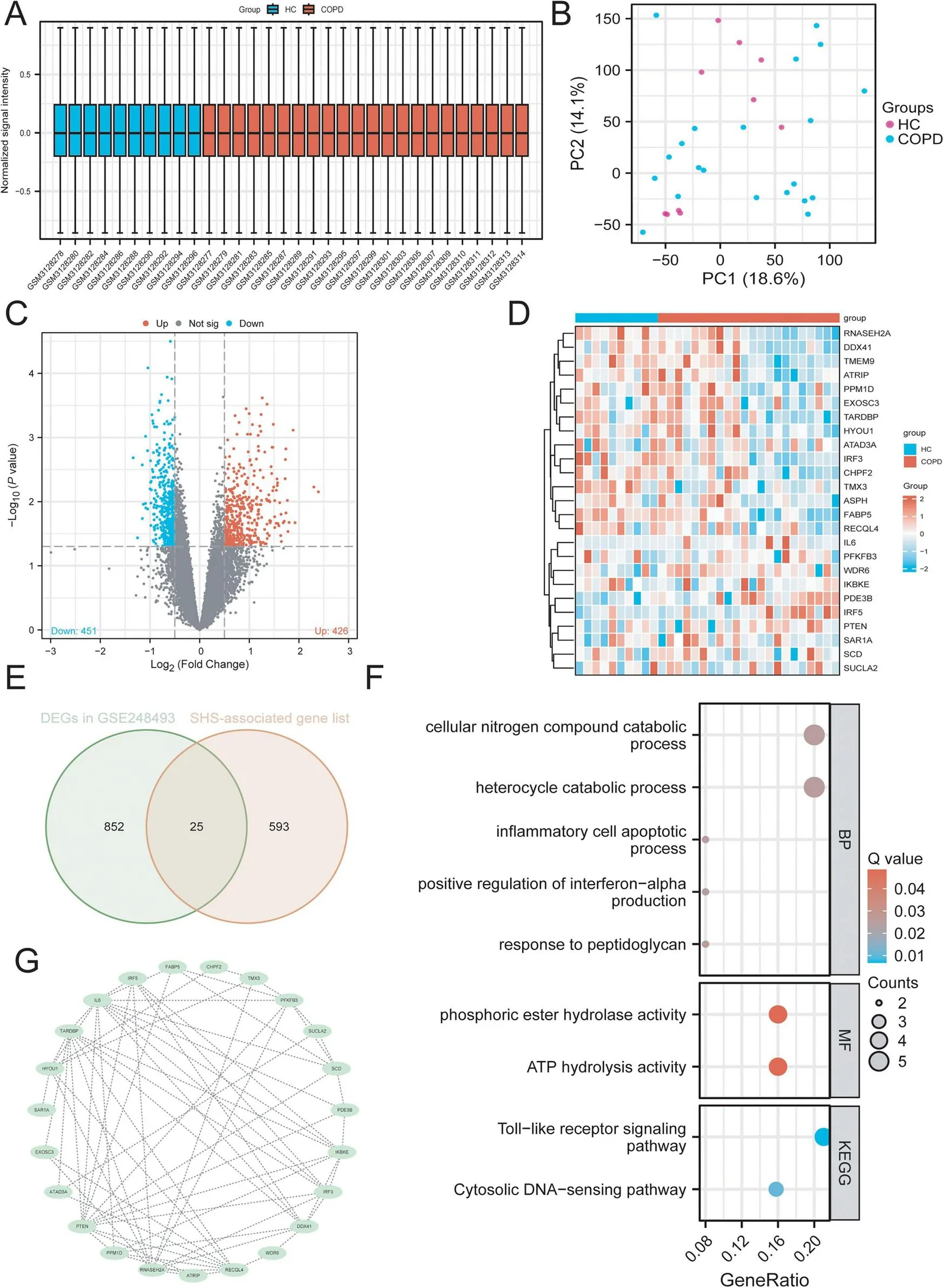
Identification of SHS-associated DEGs between COPD and HC. **(A)** Box plot of normalized samples in GSE248493. **(B)** PCA plot showing separation between groups in GSE248493. **(C)** Volcano plot of all DEGs in GSE248493. **(D)** Heatmap of the 25 SHS-associated DEGs. **(E)** Venn diagram identifying the 25 overlapping DEGs. **(F)** Bubble plot of enriched GO terms and KEGG pathways. **(G)** STRING interaction network of SHS-associated DEGs.

QC of the discovery set GSE248493 was confirmed by uniform box plots and distinct separation between HC and COPD groups in the PCA plot (Figures 2a,b). Differential expression analysis identified 877 DEGs distinguishing COPD from HC (426 up-regulated DEGs and 451 down-regulated DEGs) (Figure 2C). Intersection of these DEGs with a curated SHS-related gene list yielded 25 candidate SNS-associated DEGs (Figure 2E). A heatmap confirmed their dysregulated expression patterns (Figure 2D). KEGG and GO enrichment analyses revealed their significant involvement in pathways related to immune response and DNA repair (Figure 2F). Next, PPI network enabled the recognition of interaction patterns of SNS-associated DEGs (Figure 2G).

### 3.3 Identification of SCRP-related DEGs for COPD patients

**FIGURE 3:**
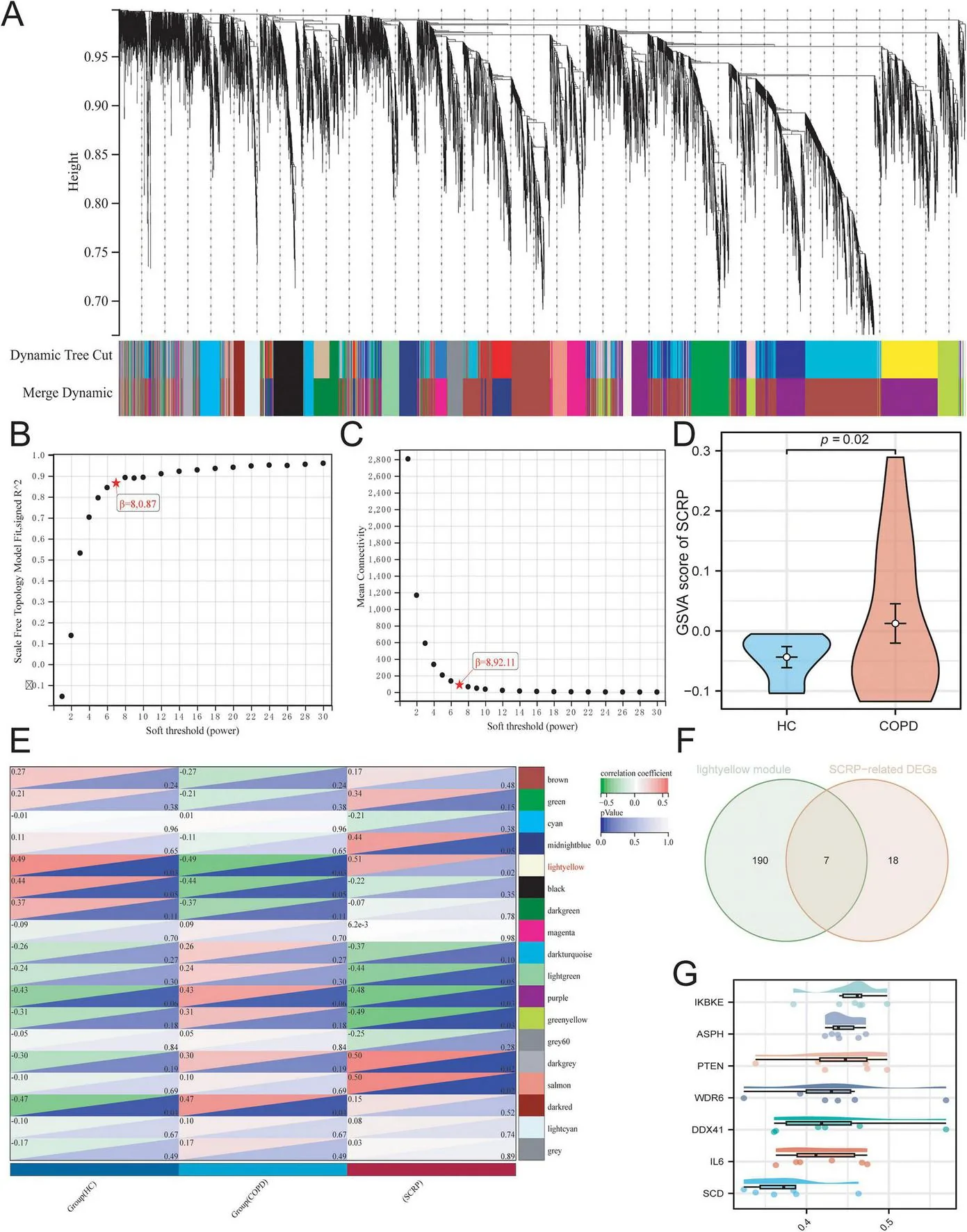
Identification of SCRP-associated shared DEGs in GSE55962. **(A)** Gene dendrogram and module assignment from WGCNA. **(B,C)** Scale-free topology fit index and mean connectivity for soft-threshold selection. **(D)** GSVA analysis comparing SCRP activity between HC and COPD groups. **(E)** Module-trait relationship heatmap highlighting the correlation of the lightyellow module with the SCRP gene set. **(F)** Venn diagram identifying the seven shared genes. **(G)** Friend analysis of the seven SS-associated DEGs.

CIBERSORT analysis of GSE55962 revealed a significant increase in SCRP level in COPD compared to HC (Figure 3D). To identify gene networks tightly linked to SCRP, we constructed a WGCNA network and identified the lightyellow module as showing the highest positive correlation with the SCRP gene set (Figures 3A–C,E). This module contained 197 candidate genes. The intersection between these SCRP-related module genes and the 25 SHS-associated DEGs pinpointed 7 core SS-associated shared genes, including IKBKE, ASPH, PTEN, WDR6, DDX41, IL6, and SCD (Figures 3F,G).

### 3.4 Identification of SS-related molecular subgroups for COPD patients

**FIGURE 4:**
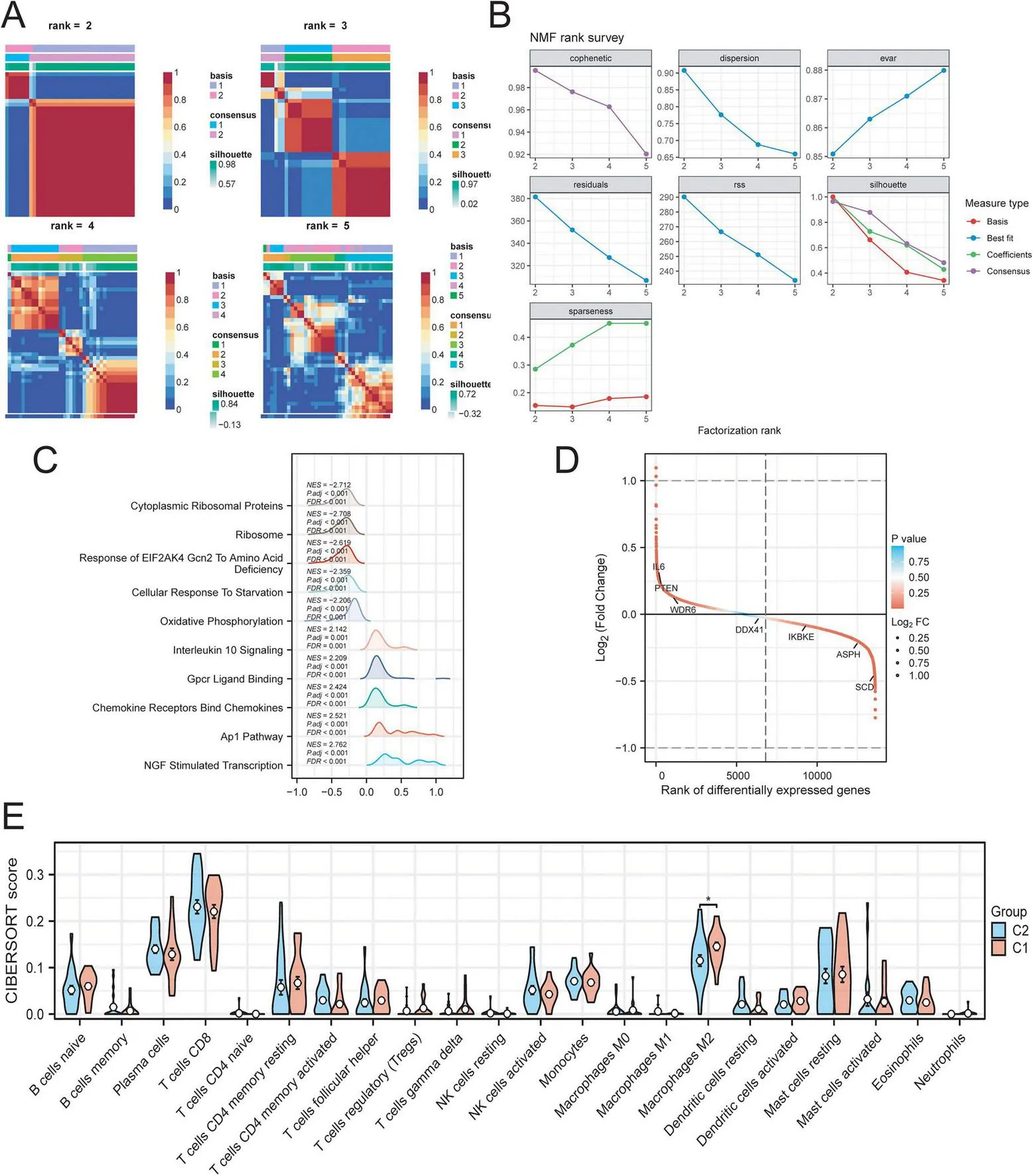
Molecular stratification of COPD patients based on the SS-associated signature in GSE77344. **(A)** Consensus clustering heatmaps for NMF ranks 2–5. **(B)** NMF rank survey metrics. **(C)** GSEA enrichment plots highlighting pathway differences between clusters. **(D)** Rank-order plot of differentially expressed genes with the seven SS-associated genes labeled. **(E)** Comparative violin plots of CIBERSORT immune cell scores between C1 and C2.

NMF consensus clustering applied to GSE77344, based on the expression of the 7 SS-associated DEGs, robustly partitioned the COPD cohort into 2 distinct molecular subtypes, designated C1 and C2 (Figures 4A,B). A heatmap confirmed differential expression of the signature genes between these subtypes (Figure 4D). GSEA indicated that C2 was enriched for pathways involved in inflammatory response and cell cycle (Figure 4C). Correspondingly, CIBERSORT analysis revealed significant differences in immune cell infiltration between the 2 clusters (Figure 4E).

### 3.5 Identification of SS-related predictive model for COPD patients

**FIGURE 5:**
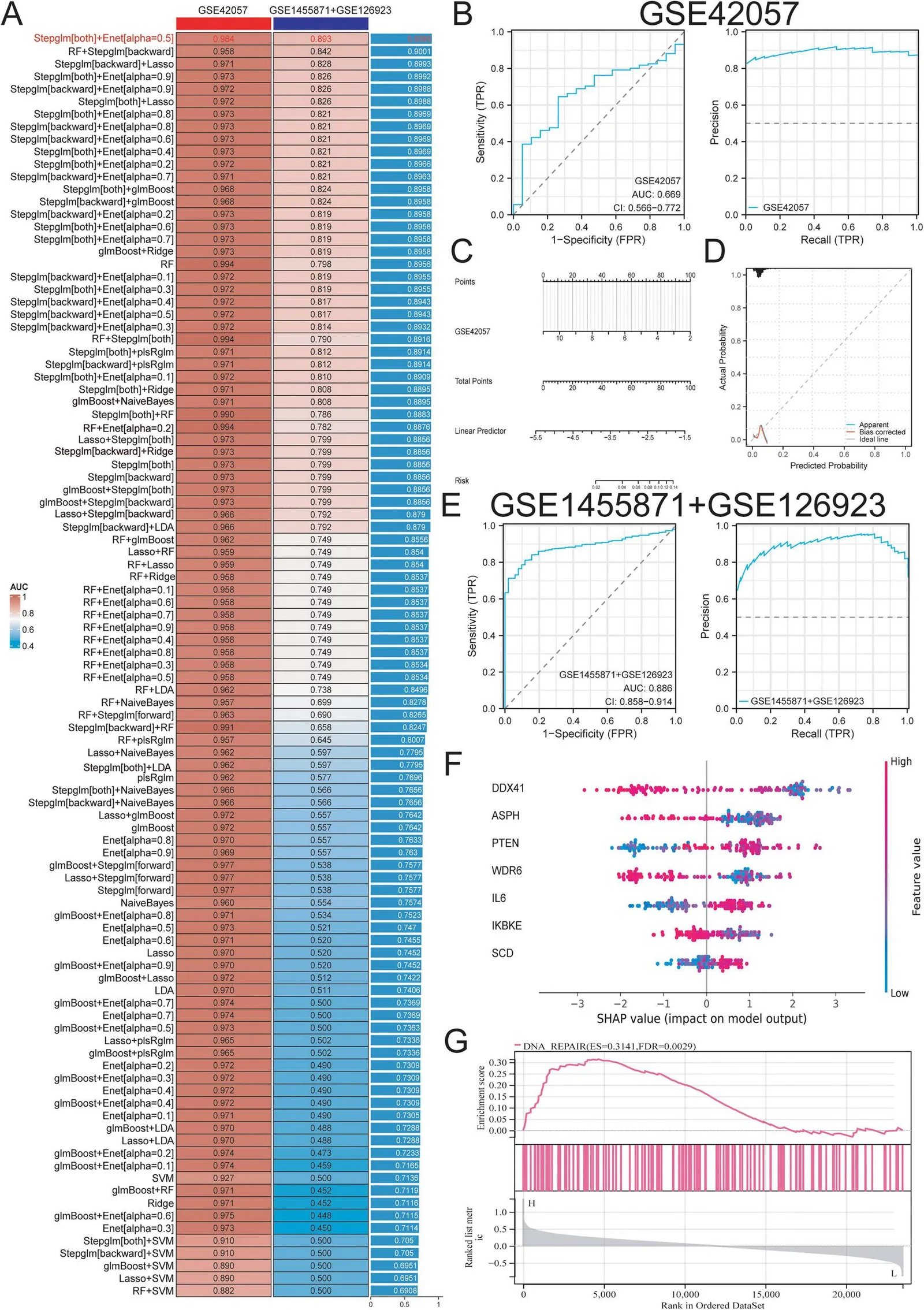
Construction and validation of the SS-associated diagnostic model. **(A)** Heatmap ranking machine-learning models by AUC across the training and validation datasets. **(B,E)** ROC and precision-recall curves in the training and validation datasets, respectively. **(C)** Nomogram for COPD risk estimation in the training set. **(D)** Calibration plot for COPD risk estimation in the training set. **(F)** SHAP summary plot. **(G)** GSEA enrichment plot for DDX41 showing a positive correlation with DNA repair.

Evaluation of the 132-model machine learning framework on the training set (GSE42057) identified a model combining Stepglm (both) with Elastic Net (Enet, alpha = 0.5) as the top performer, achieving an AUC-index of 0.9385 (Figure 5A). This model demonstrated robust diagnostic accuracy, with an AUC of 0.669 (CI = 0.566–0.772) on the training set and 0.886 on the independent validation set (CI = 0.858–0.914) (Figures 5B,E). The constructed nomogram and its calibration curve indicated excellent agreement between predicted and observed probabilities of COPD in training set (Figures 5C,D). SHAP analysis unequivocally identified DDX41 as the most influential feature contributing to the model predictive power (Figure 5F). Subsequent single-gene GSEA for DDX41 revealed a significant positive association with the DNA repair pathway (Figure 5G).

### 3.6 Single-cell analysis of COPD microenvironment

**FIGURE 6:**
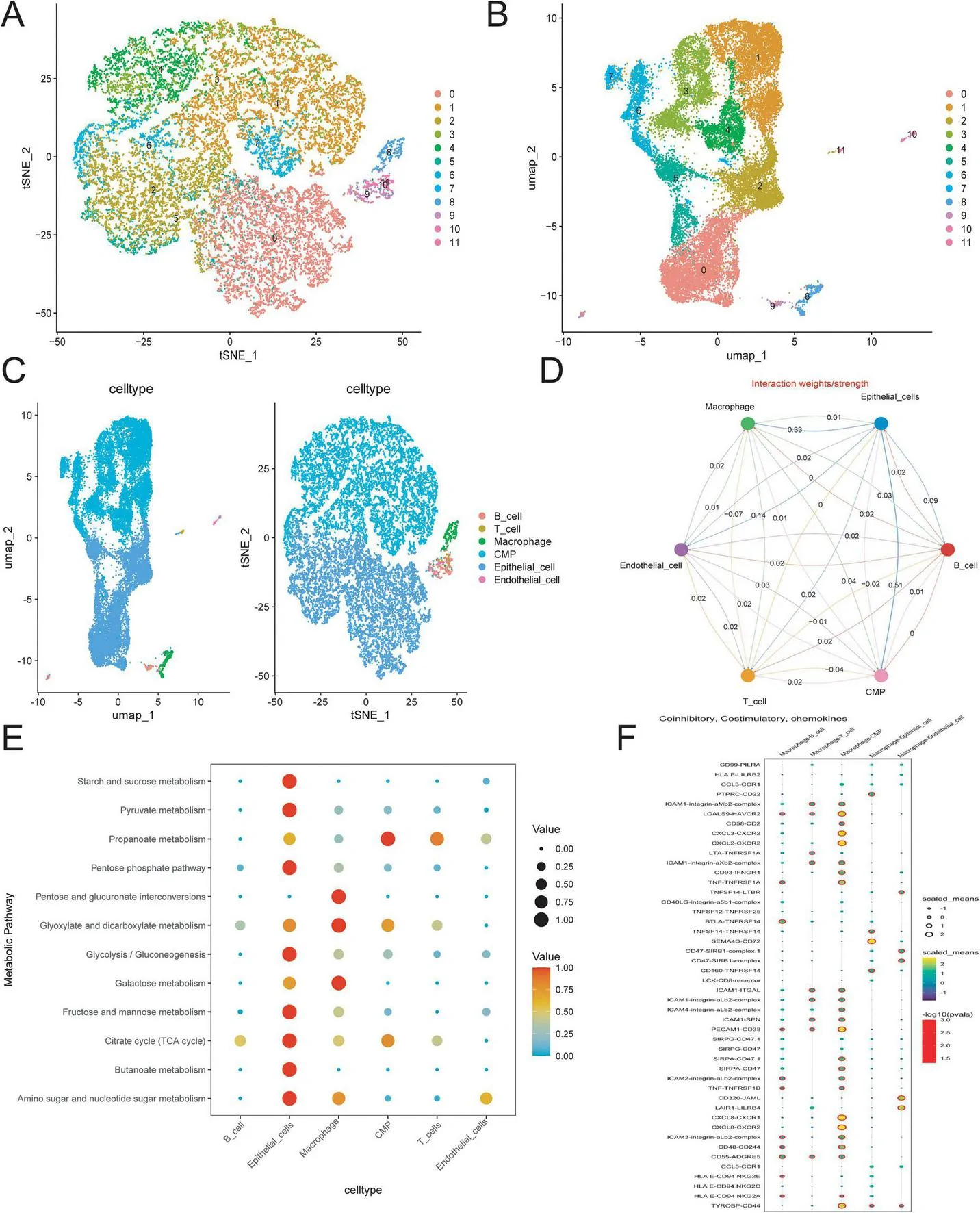
Single-cell landscape of the COPD microenvironment. **(A,B)** t-SNE and UMAP plots showing numbered cell clusters. **(C)** UMAP and t-SNE plots showing annotated cell types. **(D)** Network plot showing intercellular communication strength inferred by CellChat. **(E)** Bubble plot showing metabolic pathway activity across cell types. **(F)** Dot plot showing coinhibitory, costimulatory, and chemokine-related signals across cell types.

Analysis of the COPD scRNA-seq dataset identified 11 initial clusters, which were annotated into 6 major cell types, with T cell. B cell, macrophage, CMP, epithelial cell and endothelial cell (Figures 6A–C). CellChat analysis revealed prominent intercellular communication, with complex ligand-receptor interactions evident (Figures 6D,F). Metabolic profiling demonstrated heterogeneous activity across different cell populations (Figure 6E).

### 3.7 SS-associated hub molecular signature performance at single-cell level of COPD patients

**FIGURE 7:**
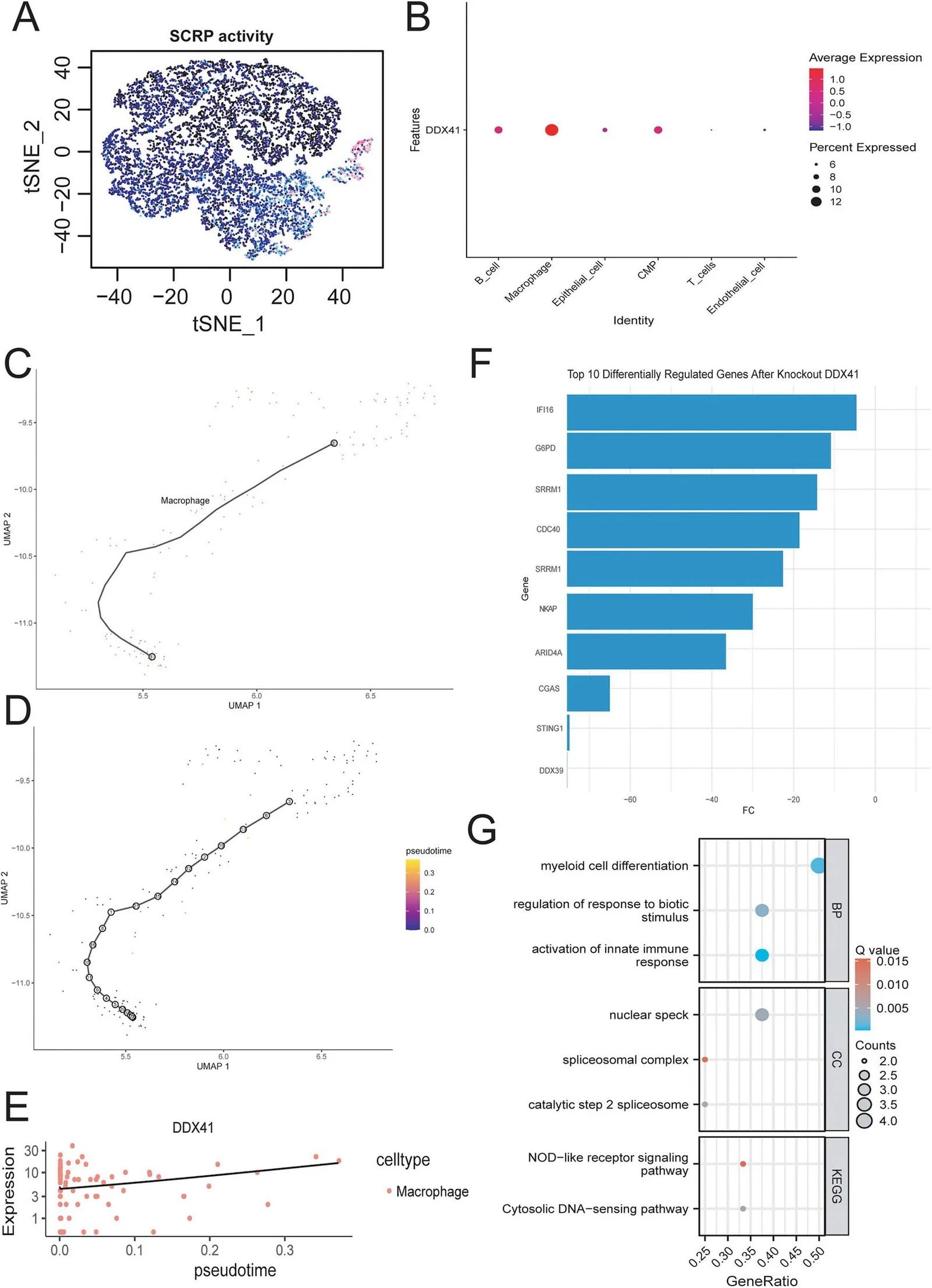
Spatial and functional resolution of the SS-associated patterns in macrophages of COPD patients. **(A)** AUCell scores of SCRP activity across cells. **(B)** Dot plot showing DDX41 expression across cell types. **(C)** UMAP trajectory of macrophages. **(D)** Macrophage trajectory colored by pseudotime. **(E)** DDX41 expression along pseudotime. **(F)** Bar plot of the top 10 differentially regulated genes after virtual DDX41 knockout in macrophages. **(G)** Enrichment analysis of perturbed genes, highlighting immune and metabolic pathways in macrophages.

AUCell scoring demonstrated that SCRP pathway activity was significantly elevated in macrophage (Figure 7A). Expression of the hub gene DDX41 was predominantly localized to and specifically expressed within the macrophage population (Figure 7B). Pseudotime trajectory analysis of macrophage revealed a continuous differentiation process, with DDX41 expression upregulating along the trajectory (Figures 7C–E). Virtual knockout of DDX41 specifically within the macrophage compartment led to widespread transcriptional perturbations (Figure 7F). Functional enrichment analysis of these perturbed genes highlighted significant involvement in immune and metabolic pathways, including NAD + metabolism and inflammatory signaling (Figure 7G).

### 3.8 Drug screening and clinical validation targeted SS-related signature for COPD patients

**FIGURE 8:**
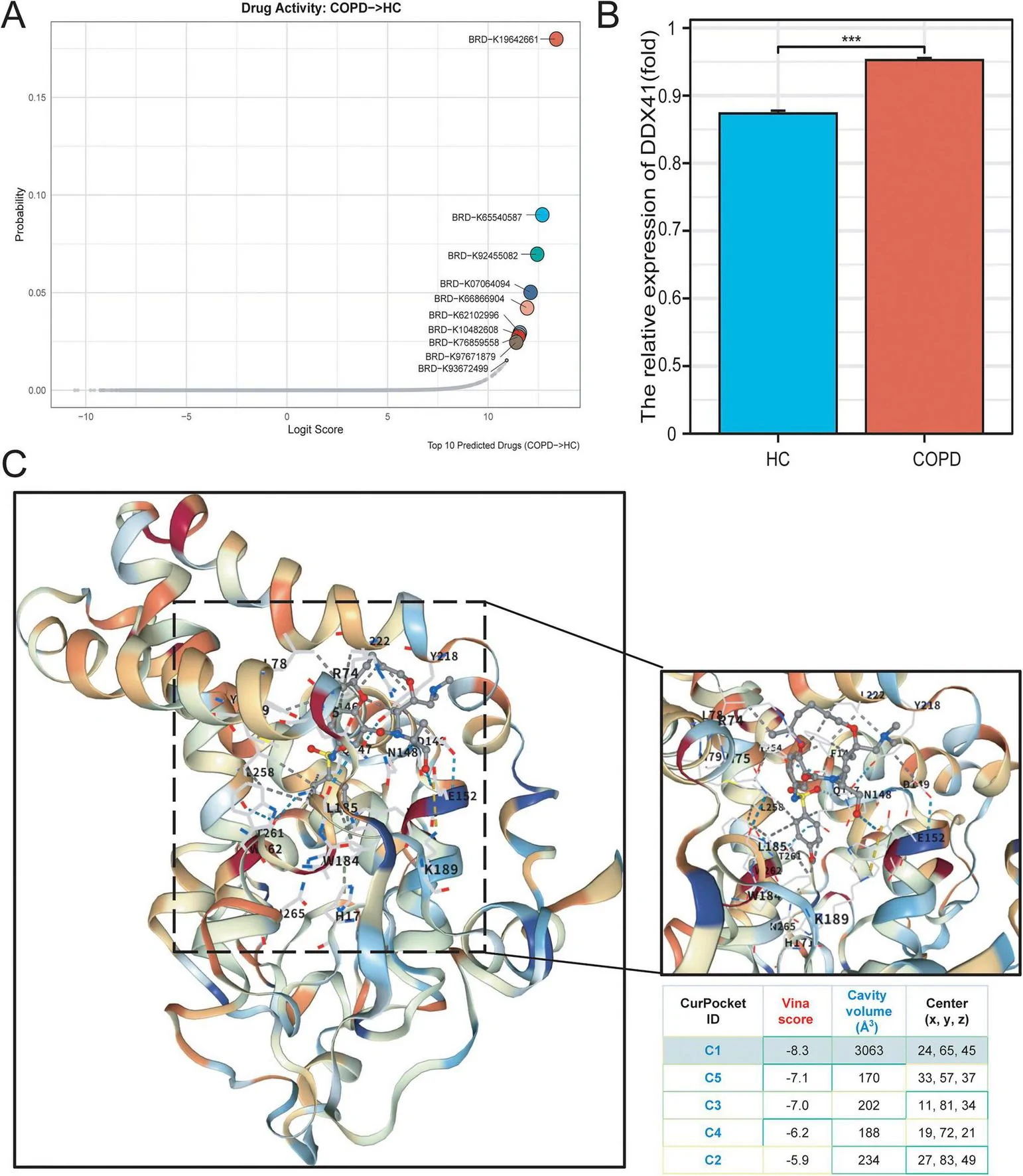
Therapeutic candidate identification and clinical validation targeting DDX41 in COPD. **(A)** Scatter plot ranking the top candidate compounds. **(B)** q-RT-PCR analysis of DDX41 expression in COPD and HC samples. **(C)** Three-dimensional representation of the molecular docking pose between BRD-K19642661 and DDX41.

Screening of compound libraries using the DrugReflector algorithm and cMAP database nominated BRD-K19642661 as the top-ranked therapeutic candidate for COPD (Figure 8A). Molecular docking simulations predicted that BRD-K19642661 binds favorably to the DDX41 protein with a calculated binding energy of -8.3 kcal/mol (Figure 8C). Finally, q-RT-PCR analysis confirmed that DDX41 mRNA expression was significantly elevated in PBMCs from patients with COPD compared to HC (Figure 8B).

## 4 Discussion and conclusion

This study presents a novel, potentially clinical actionable molecular framework linking SCRP with the pathogenesis of COPD in aging, SHS-exposed male patients. By integrating a large-scale NHANES with a multi-omics bioinformatics pipeline, we successfully decoded an SS-associated pathogenic molecular patterns in COPD. From a methodological perspective, this study has several strengths. The integration of a nationally representative cross-sectional database (NHANES) with high-resolution transcriptomic and single-cell data creates a strong translational pipeline. The use of an explainable 132-model machine learning framework ensured that the identification of DDX41 as the hub gene was robust and not an artifact of a single algorithm. Furthermore, the identification of BRD-K19642661 via AI-driven drug screening provides a testable hypothesis for future therapeutic development.

DDX41 is a member of the DEAD-box RNA helicase family and has recently gained attention for its critical role in the innate immune response, specifically as a key sensor for cytosolic DNA and an essential activator of the STING-dependent interferon pathway (50). In the context of COPD, chronic exposure to SHS is known to cause cellular stress and DNA damage within the lung microenvironment, which can trigger aberrant STING activation and a maladaptive inflammatory cascade (51). Our single-cell analysis localized DDX41 expression predominantly to macrophages and its virtual knockout perturbed gene networks related to immune and metabolic pathways, including NAD + metabolism (52). This suggests a model in which DDX41 may be involved in the transition from an acute inflammatory state to a chronic, SCRP-associated inflammatory milieu. The observation that DDX41 expression increased along the pseudotime trajectory of macrophages further indicates that its role is amplified during the differentiation and activation of these key immune effector cells, which are central to COPD pathology. Beyond its well-characterized role in cytosolic DNA sensing, emerging evidence suggests that DDX41 may intersect with several additional pathogenic mechanisms relevant to COPD. First, mitochondrial dysfunction is a hallmark of COPD pathogenesis, contributing to oxidative stress, cellular senescence, and chronic inflammation (53). Notably, recent studies have demonstrated that mitochondrial damage can trigger the release of mitochondrial DNA (mtDNA) and RNA:DNA hybrids into the cytosol, which in turn activate DDX41-STING-dependent type I interferon responses (54). This positions DDX41 as a potential molecular bridge linking SHS-induced mitochondrial injury to the sustained inflammatory state characteristic of COPD (55). Second, eosinophil extracellular trap cell death (EETosis) has been identified as a pathogenic process in COPD, particularly in the eosinophilic subtype which accounts for 20–40% of COPD patients (56). EETosis is a deliberate form of cell death by which eosinophils release extracellular traps, and its debris may trigger uncontrolled NETosis and perpetuate airway inflammation (56). While a direct link between DDX41 and EETosis has not yet been established, DDX41 role in regulating nucleic acid-sensing and type I interferon responses raises the hypothesis that it may modulate eosinophil activation or the inflammatory milieu that promotes EETosis (56). Third, DDX41 has been shown to regulate R-loop metabolism and m6A RNA methylation through its interaction with the m6A-METTL complex (MAC) and YTHDC1 (57). DDX41 deficiency disrupts METTL3-YTHDC1 complex assembly, leading to R-loop accumulation and genomic instability (57). Given that m6A modification plays a critical role in regulating inflammatory gene expression and innate immune responses, DDX41 may influence COPD pathogenesis through epigenetic mechanisms that modulate macrophage activation and cytokine production (57).

In conclusion, this study suggests a novel conceptual link between a common clinical biomarker (SCRP) and a defined molecular axis (DDX41-mediated innate immunity) in aging, SHS-exposed male COPD patients. Our findings position DDX41 as a promising diagnostic biomarker and a potential therapeutic target. Despite these strengths, several limitations must be acknowledged. First, COPD status in the NHANES cohort was determined using self-reported physician diagnosis rather than spirometry-confirmed airflow obstruction. While this approach has been used in previous NHANES-based COPD studies and demonstrates reasonable validity for population-level prevalence estimation, it may introduce misclassification bias. We acknowledge that the absence of spirometric data limits the diagnostic precision of our epidemiological findings and may affect the generalizability of the observed associations. Second. The foundational transcriptomic insights are derived from COPD publicly available datasets, which, despite batch correction and without definite information with SHS exposure, may not fully account for all sources of technical and biological variability. Future studies should combine the clinical information of COPD patients, such as CRP level, SHS exposure, age and sex, focusing on the estimation of efficacy of diagnostic and molecular stratification models acquired from our study in a large, multi-center clinical trials to enhance the clinical significance. Besides, The single-cell analysis, while offering high-resolution insights, is based on a cohort of 3 COPD patients, potentially limiting the capture of the full heterogeneity of SHS states across the COPD spectrum. Future studies should combined advanced AI technologies in higher resolution single-cell and spatial data of COPD patients, coupled with pre-clinical studies, dynamically tracing SS-associated patterns for the pathogenesis of COPD patients. In addition, the precise molecular cascades downstream of DDX41 in macrophage that ultimately impair COPD patient health caused by SHS remaining unclear. Future research should prioritize *in vivo* studies using conditional knockout or macrophage-specific overexpression of DDX41 in COPD mouse models to establish causality and elucidate detailed mechanisms acquired by our *in silico* knockout. Concurrently, high-throughput screening for macrophage-selective DDX41 modulators could yield more targeted therapeutic leads than existing pan-inhibitors. Similarly, the therapeutic candidate, BRD-K19642661, identified computationally, demands rigorous experimental testing in preclinical models to confirm its efficacy, optimal dosing, and ability to penetrate the blood barrier and modulate the proposed SHS-associated molecular patterns and DDX41 in the treatment of COPD. Finally, the q-RT-PCR validation cohort comprised only 8 COPD patients and 8 healthy controls. This small sample size limits the statistical power to detect modest expression differences and precludes assessment of biomarker performance metrics such as sensitivity and specificity. Additionally, the small cohort may not capture the full heterogeneity of COPD, particularly across different disease severities, ages, and smoking histories. Future studies with larger, prospectively collected cohorts are required to confirm DDX41 as a robust clinical biomarker.

## Data Availability

The datasets presented in this study can be found in online repositories. The names of the repository/repositories and accession number(s) can be found in the article/Supplementary material.
